# BRCA1-Associated Protein 1 in Sarcoma

**DOI:** 10.3390/cancers18213496

**Published:** 2026-10-30

**Authors:** Xingliang Liu, William Haugh, Ziqiang Zhang, Jianguo Huang

**Affiliations:** 1 Earle A. Chiles Research Institute, Providence Cancer Institute, Portland, OR 97213, USA; 2 Department of Respiratory and Critical Care Medicine, Shanghai Pudong Hospital, Fudan University Pudong Medical Center, Pudong Hospital of Fudan University, Shanghai 201399, China

**Keywords:** STS, BAP1, PR-DUB, PLK1, therapeutic target

## Abstract

Soft tissue sarcomas (STSs) are genetically complex mesenchymal tumors with limited therapeutic options. BRCA1-associated protein 1 (BAP1), a deubiquitinase, is a critical regulator of chromatin architecture, DNA repair, and cell cycle control. While *BAP1* alterations are well-characterized in mesothelioma and uveal melanoma, their role in STS remains understudied. Increasing evidence shows *BAP1* alterations are associated with STS. This review summarizes current evidence on BAP1 biology and its emerging significance in sarcoma. We discuss BAP1’s molecular functions, including its role in the Polycomb Repressive Deubiquitinase (PR-DUB) complex, and summarize recent findings implicating *BAP1* loss in multiple subtypes of STS. We highlight mechanistic links between *BAP1* deficiency, genomic instability, and tumor progression, and explore therapeutic implications such as polo-like kinase 1 (PLK1) inhibition and epigenetic modulation. Understanding *BAP1*-driven pathways in STS may uncover novel biomarkers and targeted strategies, addressing a critical unmet need in sarcoma management.

## Simple Summary

Soft tissue sarcomas are a diverse group of rare cancers with limited treatment options, and their underlying molecular causes remain incompletely understood. BRCA1-associated protein 1 (BAP1) is a tumor suppressor involved in chromatin regulation, DNA repair, and other cellular processes. Although *BAP1* alterations are well established in several cancers, their significance in sarcoma has received less attention. This review summarizes current evidence linking *BAP1* alterations to different sarcoma subtypes, including findings from genetic, clinical, and functional studies. We also discuss how *BAP1* loss may influence sarcoma development, tumor progression, and the tumor immune environment, as well as potential therapeutic opportunities associated with *BAP1* deficiency. Further studies are needed to define the clinical significance of germline or somatic *BAP1* alterations and determine whether *BAP1* deficiency can serve as a biomarker or therapeutic vulnerability in specific sarcoma subtypes.

## 1. Introduction

Soft tissue sarcomas (STSs) represent a heterogeneous group of mesenchymal malignancies characterized by diverse histologic subtypes and variable clinical behavior [1]. Despite advances in surgical techniques, radiotherapy, and chemotherapy, the prognosis for patients with advanced or metastatic STS remains poor, underscoring the need for an improved molecular understanding and targeted interventions [2].

BRCA1-associated protein 1 (BAP1) has evolved from a BRCA1-interacting deubiquitylase into one of the most intensively studied tumor suppressors at the intersection of chromatin regulation, cell-state control, DNA repair, and cancer susceptibility. A previous study established BAP1 as a nuclear ubiquitin carboxy-terminal hydrolase that binds BRCA1, but the field changed decisively once recurrent loss-of-function alterations were recognized in uveal melanoma, malignant mesothelioma, renal cell carcinoma, cholangiocarcinoma, and several other malignancies and once germline *BAP1* pathogenic variants were shown to define a hereditary cancer syndrome now referred to as BAP1 tumor predisposition syndrome (BAP1-TPDS) [3,4,5]. Furthermore, our recent study identified *BAP1* loss as a functional driver of sarcoma development, providing direct evidence for a tumor-suppressive role of *BAP1* in sarcomagenesis [6]. The loss of BAP1 function may contribute to genomic instability and epigenetic dysregulation, fostering tumor development and progression. However, its precise biological and clinical significance in STS remains incompletely understood.

This review summarizes the current knowledge of BAP1 biology and its emerging role in STS, with an emphasis on molecular mechanisms and potential therapeutic implications. We further highlight mounting evidence linking *BAP1* alterations to sarcoma development and suggest that sarcoma should receive greater attention in the broader BAP1 research field rather than being overlooked because of its rarity and heterogeneity. By integrating genomic, clinical, and functional evidence, we aim to stimulate further investigation of *BAP1*-deficient sarcoma and its potential clinical significance.

## 2. Epidemiology and Clinical Challenges of Soft Tissue Sarcoma

STS comprises a heterogeneous group of rare mesenchymal malignancies, accounting for less than 1% of adult cancers and up to 7% of cancers in children and adolescents younger than 20 years [7,8,9]. Globally, STS remains an uncommon cancer, with incidence estimates generally near ~1–2 cases per 100,000 person-years across populations. In the United States, the American Cancer Society projects ~13,910 new STS cases and ~5400 deaths in 2026, representing ~0.7% of all new cancer diagnoses and ~0.9% of all cancer deaths [10]. These estimates refer to soft-tissue malignancies and do not include primary bone sarcomas, which are reported separately. The SEER Program reports an aggregate five-year relative survival of approximately 65.7% for the registry category “soft tissue including heart cancer” among patients diagnosed during 2016–2022. This population-level estimate should be interpreted cautiously because survival varies substantially according to the histologic subtype, anatomic site, age, and tumor grade.

STS comprises more than 75 histologic subtypes, classified by the tissue of origin and molecular features [11,12]. Common adult subtypes include undifferentiated pleomorphic sarcoma (UPS), liposarcoma (well-differentiated, dedifferentiated, myxoid, pleomorphic), leiomyosarcoma, synovial sarcoma, malignant peripheral nerve sheath tumor (MPNST), angiosarcoma, epithelioid sarcoma, etc. Gastrointestinal stromal tumor (GIST) may not be consistently represented in this site-based category because tumors arising in gastrointestinal organs can be classified under their corresponding primary anatomic sites [10]. Pediatric-predominant subtypes include rhabdomyosarcoma (RMS), desmoplastic small round cell tumor, etc.

The low incidence and diverse histology hinder large-scale trials in STS. STS often presents as painless masses, leading to delayed detection and an advanced stage at diagnosis. Furthermore, despite optimal local control, up to 40–50% of patients with STS develop metastatic diseases, primarily to the lungs [13,14]. Conventional chemotherapy has remained unchanged for more than four decades; for instance, first-line therapy remains as anthracycline-based chemotherapy [15]. Targeted therapies are effective only in a small subset of STS [16,17]. Overall, the development of targeted therapies for STS lags behind other common cancers. The main reason is that the molecular pathogenesis of STS remains largely unknown, hindering the development of effective targeted therapies for STS driven by specific oncogenic drivers or tumor suppressor drivers, such as *BAP1*.

## 3. BRCA1-Associated Protein 1

### 3.1. Domain Structure, Conservation, and Molecular Organization

**Figure 1:**
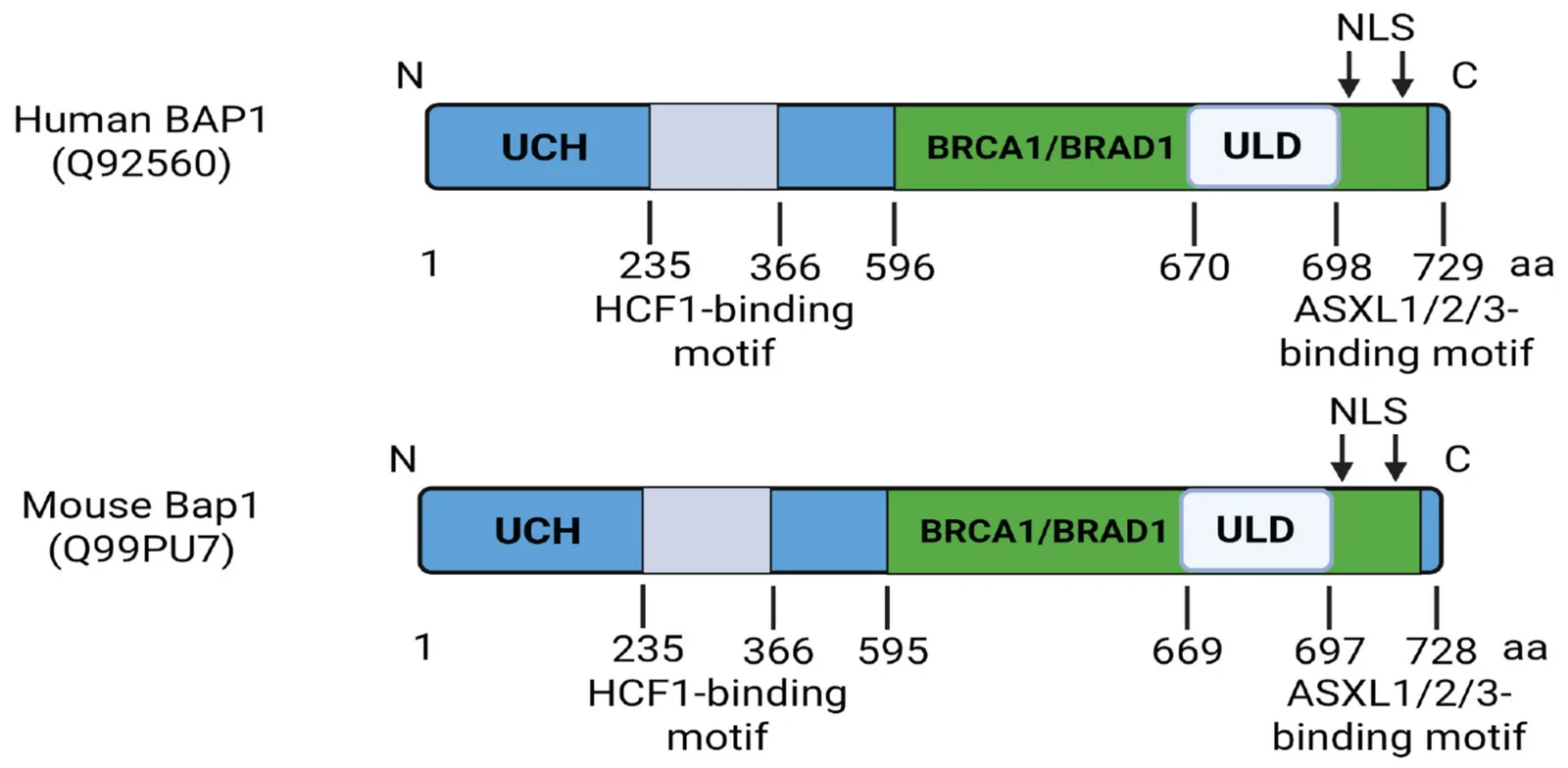
Comparative structural organization of BAP1 across species. Both proteins contain an N-terminal ubiquitin C-terminal hydrolase (UCH) catalytic domain (human and mouse: aa 4–235), an HCF1-binding motif (human and mouse: aa 363–366), a BRCA1/BRAD1-binding region (human: aa 596–721; mouse: aa 595–720), and a UCH37-like domain (ULD) at the C-terminus (human: aa 670–698; mouse: aa 669–697). Additional conserved features include ASXL1/2/3 interaction sites within the BRCA1-binding region and nuclear localization signals (NLSs) near the C-terminal.

BAP1 is highly conserved among vertebrates, with Xenopus Bap1 sharing 71% amino acid identity with human BAP1 and zebrafish Bap1 sharing 66% identity; notably, amino acid identity exceeds 90% within conserved functional regions, including the catalytic domain and binding motif [18,19]. For instance, human and mouse BAP1 share ~98–99% sequence identity, indicating the near-complete conservation of functional domains. Figure 1 compares the main functional domains in both the human and mouse BAP1 protein. Human (UniProtKB: Q92560; 729 amino acids; Ensembl gene ID: ENSG00000163930) and mouse (UniProtKB: Q99PU7; 728 amino acids; Ensembl gene ID: ENSMUSG00000021901) BAP1 share a highly conserved domain architecture, which is also broadly maintained across vertebrates [18,20,21,22,23,24,25,26,27]. From N-terminal to C-terminal, both human and mouse BAP1 contain a ubiquitin C-terminal hydrolase (UCH) catalytic domain, a breast cancer gene 1 (BRCA1)-binding region, a UCH37-like domain (ULD), and nuclear localization signals (NLS). Additionally, both human and mouse BAP1 contain an HCF1-binding motif and additional sex combs-like 1/2/3 (ASXL1/2/3) interaction sites within the BRCA1-binding region. Although canine BAP1 is less extensively annotated, two protein entries in dog (*Canis lupus familiaris*), UniProtKB A0A8I3SCQ2 (752 amino acids) and A0A8I3QAS2 (729 amino acids), both contain an annotated UCH domain spanning residues 4–235, consistent with the conservation of this catalytic region. We mention canine *BAP1* because spontaneously occurring sarcomas in dogs provide an established comparative oncology model for investigating sarcoma biology and developing immunotherapies. Canine STSs share histopathological features with human STSs [28], and comparative immune profiling has identified gene-expression similarities between canine UPS and human UPS [29]. Canine sarcoma models have also supported the development of B7-H3-directed CAR T-cell approaches [30]. Further investigation is needed to establish a comprehensive annotation of canine *BAP1*, including its transcript variants and the domain organization of the encoded proteins.

Initially, BAP1 was identified as a binding partner of BRCA1 [18]. Indeed, multiple studies showed that BAP1 is involved in double-stranded DNA repair by interacting with BRCA1/RCA1-associated RING domain protein 1 (BRAD1), the disruption of which results in genome instability and cancer cell progression [18,20,21,31,32,33]. Interestingly, Ventii KH showed evidence that BAP1 can suppress tumor growth independently of BRCA1 [34]. However, whether this effect also occurs independently of BARD1 was not established in that study. Instead, its deubiquitinating enzyme is critical for its tumor-suppressor function. For instance, a study showed that BAP1 can regulate the Hippo pathway by deubiquitinating and stabilizing Large Tumor Suppressor Kinase (LATS) [35].

### 3.2. PR-DUB Complex and Chromatin Regulation

**Figure 2:**
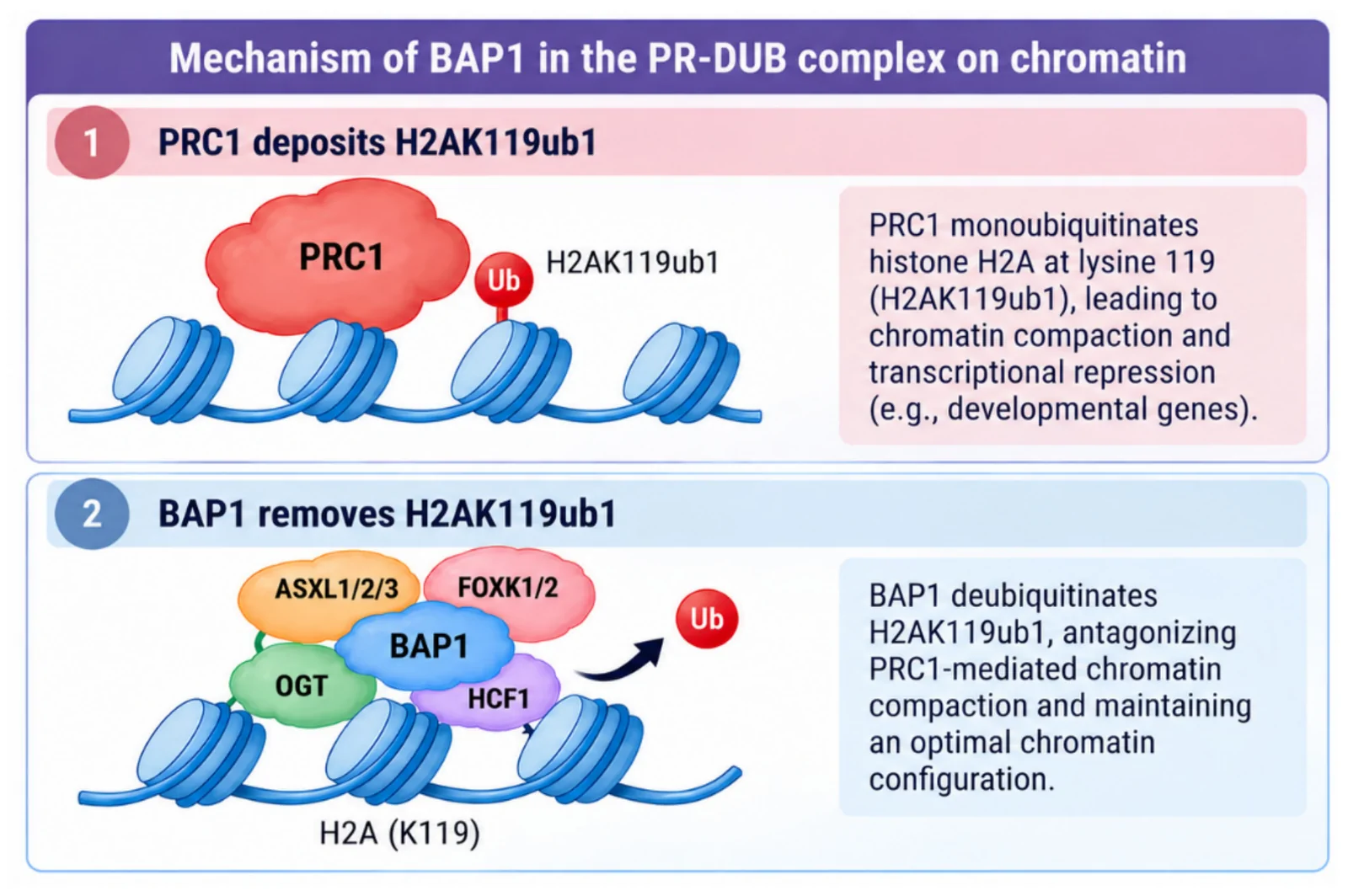
Mechanism of BAP1-mediated H2AK119 deubiquitination by the PR-DUB complex. PRC1 monoubiquitinates histone H2A at lysine 119 (H2AK119ub1), promoting chromatin compaction and transcriptional repression. In the PR-DUB complex, BAP1 associates with ASXL1/2/3, FOXK1/2, OGT, and HCF1 and removes ubiquitin from H2AK119ub1. This activity counteracts excessive PRC1-mediated H2AK119ub1 accumulation and helps maintain an optimal chromatin configuration and appropriate gene regulation.

A key lesson from the past decade is that BAP1 should not be viewed as an isolated deubiquitinase. Instead, it functions through context-dependent multiprotein assemblies. For instance, BAP1 can epigenetically regulate the chromosome by removing monobiquitin from histone H2A at lysine 119 (H2AK119ub1) via the Polycomb Repressive deubiquitinase (PR-DUB) complex. This complex was first identified in Drosophila in 2010 [36]. The core complex of PR-DUB complex in Drosophila contains Calypso (ortholog of mammalian BAP1) and Additional sex combs (ASX) (ortholog of mammalian ASXL proteins). This complex specifically removes H2AK119ub1, a mark deposited by Polycomb repressive complex 1 (PRC1). They also found that this complex is essential for proper developmental gene silencing in Drosophila. Additionally, mutations in Calypso or ASX disrupt Polycomb group (PcG)-mediated repression, leading to homeotic transformation. Similarly, in mammals, the PR-DUB complex comprises BAP1, Additional ASXL1/2/3, Forkhead Box K1/2 (FOXK1/2), O-linked N-Acetylglucosamine Transferase (OGT), and Host Cell Factor 1(HCF1) (Figure 2). Interestingly, a study showed that BAP1 recruits the PR-DUB complex to sites of DNA damage to promote the repair of DNA double-strand breaks and enhance cell survival upon DNA damage [33]. To investigate how this complex epigenetically regulates the genome, Foglizzo M et al. examined the crystal structure of the Drosophila PR-DUB and found that the deubiquitinase Calypso and its partner ASX form a 2:2 complex, the disruption of which has no impact on catalytic activity but inhibits the removal of H2AK119Ub, suggesting that high local concentrations drive assembly of the bidentate PR-DUB complex on chromatin to enhance PR-DUB activity at specific genomic loci [37]. However, these structural and biochemical findings do not establish that mammalian BAP1-ASXL complexes adopt the same 2:2 bidentate architecture or require this assembly for chromatin-associated activity. Conservation of the H2A deubiquitinase function should therefore be distinguished from the conservation of complex architecture. Rather than constituting a single invariant complex, mammalian PR-DUB comprises alternative BAP1-ASXL assemblies associated with regulatory factors such as FOXK1/2, OGT, and HCF1. BAP1 forms mutually exclusive complexes with ASXL1 or ASXL2 [24], whereas ASXL3 can bridge BAP1 to BRD4 in achaete-scute family bHLH transcription factor 1 (ASCL1)-high small-cell lung cancer [27]. Kolovos et al. demonstrated the ASXL- and FOXK1/2-dependent recruitment of BAP1 to chromatin and the maintenance of target-gene expression [38]. Ge W et al. and Thomas JF et al. found that the molecular interactions of BAP1 and ASXL1 with histones and DNA are critical for remodeling the nucleosome and H2AK119Ub by investigating the crystal structure of the PR-DUB complex in human cells [39,40]. Through chromatin immunoprecipitation followed by sequencing (ChIP-seq), they further found that there are minimal overlap and functional interactions between BAP1 and PRC2 in embryonic stem cells, challenging the previous paradigm that PR-DUB cooperates with PRCs in epigenetic gene regulation. However, this does not exclude indirect coupling through H2AK119ub1 regulation. Conway et al. showed that BAP1 loss in mouse embryonic stem cells causes widespread H2AK119ub1 accumulation, PRC2 redistribution, and diffuse H3K27me3 accumulation accompanied by increased global chromatin condensation. Nevertheless, the redistribution of PRC2 away from canonical Polycomb targets weakens its repression, permitting inappropriate target-gene activation [41]. In Drosophila, Bonnet et al. demonstrated that excessive H2Aub1 following PR-DUB catalytic loss increases accessibility at Polycomb targets and impairs nucleosome stacking and chromatin-fiber folding in vitro, resulting in target-gene derepression despite retained H3K27me3 and Polycomb binding [42]. Thus, both studies support a role for PR-DUB in maintaining Polycomb target-gene silencing, although they identify distinct mechanisms and consequences for chromatin organization, though mounting evidence demonstrates the critical role of BAP1 in cell biology and tumor biology through the PR-DUB complex. However, the precise mechanisms by which BAP1 regulates the normal cell function and tumorigenesis remains largely unknown, warranting further investigations.

## 4. BAP1 Alterations in Cancer

### 4.1. Pan-Cancer Prevalence and Alteration Types

In the largest recent The Cancer Genome Atlas (TCGA)-based re-analysis, Sturgill et al. used local realignment and multi-caller integration to increase somatic *BAP1* mutation detection by 41%, expanding the mutation catalog from 182 to 257 variants, including previously undercalled long indels. Even so, coding mutations remain only part of the picture [5]. Across TCGA, copy-number loss is the dominant alteration mechanism, with 1547 tumors showing *BAP1* copy-number loss. Copy-number loss refers to genomic loss involving the *BAP1* locus, which can reduce *BAP1* expression and consequently contribute to the loss of BAP1 function. When mutation and copy-number events are combined, alterations are especially prominent in kidney renal clear cell carcinoma, cholangiocarcinoma, uveal melanoma, mesothelioma, lung squamous carcinoma, and head and neck squamous carcinoma, each exceeding 30% of altered samples in that dataset.

This pan-cancer perspective is critical because it reframes how BAP1 biology should be interrogated in solid tumors. If one focuses only on truncating or missense variants, many tumor types appear to be “BAP1-silent”. However, once copy-number loss and expression consequences are included, *BAP1* disruption becomes much more widespread [5]. Thus, *BAP1*-deficient biology cannot be defined solely by the mutation status, an important consideration for biomarker development and for sarcoma, where complex copy-number alterations are common. Nevertheless, *BAP1* copy-number loss should be interpreted cautiously: such events may represent single-copy loss rather than complete BAP1 inactivation and can occur as part of broader chromosome 3p deletions involving additional tumor suppressor genes. Therefore, although *BAP1* copy-number loss is associated with reduced BAP1 expression, copy-number loss alone does not establish BAP1-specific functional inactivation or causality.

### 4.2. Germline vs. Somatic BAP1 Alterations

The hereditary counterpart of sporadic *BAP1* loss is BAP1-TPDS, an autosomal dominant cancer predisposition syndrome defined by a heterozygous germline pathogenic or likely pathogenic *BAP1* variant. The first report ever linking *BAP1* germline mutations causing cancer traces back to 15 years ago [4]. By investigating two mesothelioma families, Testa JR et al. found that those patients with mesothelioma all share germline mutations in *BAP1* while none of them have been exposed to asbestos, a chemical that can cause mesothelioma. Therefore, they identified the existence of a BAP1-related cancer syndrome characterized by mesothelioma and uveal melanoma, among others. Interestingly, they also found that one female patient with germline mutations in *BAP1* developed leiomyosarcoma, the first evidence that germline mutations in *BAP1* can result in sarcoma [4]. In the same year, Wiesner et al. demonstrated that inherited *BAP1* mutations predispose the individual to distinctive melanocytic neoplasms and melanoma [4,43]. Clinical studies suggest that germline BAP1-associated tumors may display less aggressive behavior than cancers with somatic *BAP1* loss. For instance, uveal melanomas arising in patients with germline *BAP1* mutations exhibit a lower risk of metastasis and a more favorable prognosis than tumors with somatic *BAP1* mutations, suggesting distinct biological consequences of inherited versus acquired *BAP1* loss [19,44]. However, the small germline subgroup and potential ascertainment, surveillance, follow-up, and treatment-era differences limit a causal interpretation. Prospective, multicenter studies accounting for the tumor stage, molecular risk features, surveillance, and treatment are needed to establish whether the germline status independently influences the outcome. Although BAP1 regulates lineage commitment and differentiation-associated chromatin states, whether the timing of biallelic inactivation or the surrounding tissue context explains the reported clinical difference remains unknown. This interesting observation is still unknown, further suggesting that the precise mechanisms by which BAP1 regulates the normal cell function and tumorigenesis warrant further investigations.

Interestingly, the phenotypic consequences of germline *BAP1* alterations may extend beyond cancer predisposition. Küry et al. identified rare de novo heterozygous missense variants in *BAP1* associated with a syndromic neurodevelopmental disorder, now referred to as Küry–Isidor syndrome [45,46]. The reported phenotype was variable and included developmental and speech delay, behavioral abnormalities, and, in some individuals, seizures and other systemic manifestations. These findings broaden the spectrum of human disorders associated with germline *BAP1* alterations and suggest that different classes of *BAP1* variants may have distinct developmental and tumor-predisposition consequences. Further studies are needed to define the genotype–phenotype relationships that determine these divergent clinical manifestations. From a genotype–phenotype perspective, Küry–Isidor syndrome is associated with specific de novo heterozygous missense variants, whereas BAP1 tumor predisposition syndrome encompasses truncating, splice-site, and pathogenic missense variants. Several Küry–Isidor-associated variants impair H2A deubiquitination and alter chromatin regulation, linking developmental dysfunction to molecular processes also relevant to cancer. However, these findings do not establish equivalent tumor-predisposing effects. For sarcoma research, the distinction underscores the need for a variant-specific functional assessment rather than assuming that all pathogenic germline *BAP1* variants confer the same cancer risk.

## 5. BAP1 in Sarcoma

The majority of STS is dominated, in many subtypes, by structural variation, copy-number complexity, and tumor suppressor loss rather than by a small set of shared activating oncogene mutations [1,47]. That general feature makes BAP1 particularly interesting: it is exactly the kind of lesion that could be biologically central yet underappreciated if one relies on mutation-frequency rankings alone. For instance, the TCGA pan-cancer datasets do not identify sarcoma as one of the top *BAP1*-altered lineages in the same way that they identify uveal melanoma or mesothelioma. However, Sturgill et al.’s analysis makes a broader point that is directly relevant to STS: once copy-number loss and expression consequences are considered, functionally meaningful *BAP1* disruption becomes easier to detect than that with coding mutations alone [5].

A recurring limitation in the BAP1 cancer literature is that sarcoma is frequently not presented as a distinct disease category but is instead grouped under broad labels such as “other cancers” or “other rare cancers” [4,48,49]. This convention reflects the relative rarity and histologic heterogeneity of STS, as well as the fact that *BAP1* alterations are most prominently recognized in uveal melanoma, mesothelioma, renal cell carcinoma, and cholangiocarcinoma. However, placing STS in an “other” category may inadvertently minimize its biological relevance and obscure subtype-specific associations, particularly in complex-karyotype sarcomas where copy-number loss, epigenetic dysregulation, and tumor suppressor cooperation may be more informative than the point mutation frequency alone. Therefore, STS should be evaluated explicitly in BAP1-focused studies rather than treated as a residual category, especially as emerging germline, genomic, and functional evidence supports a role for BAP1 loss in sarcomagenesis [4].

### 5.1. BAP1 Alterations in Sarcoma

Testa et al. reported one female patient with germline *BAP1* mutations (c.1717del) who developed leiomyosarcoma, positioning leiomyosarcoma as an early example of sarcoma appearing among non-core/other BAP1-associated cancers (Table 1) [4]. The first report of a *BAP1* mutation in bone sarcoma, e.g., osteosarcoma, was in 2017, when Engert F et al. showed one human osteosarcoma cell lines bears a mutation in *BAP1* [50]. Another possible reason why *BAP1*-mutant sarcoma is understudied is that sarcoma is a rare tumor type that is often missed or misdiagnosed as other cancers. For instance, a first report showing the first type of tumor detected in a patient with BAP1-TPDS was high grade MPNST, which was the first reported case showing that BAP1-TPDS can be associated with MPNST. This case also led to a diagnosis of the patient’s family of being associated with BAP1-TPDS (pathogenic variant in *BAP1* (c.659 + 1delG (IVS8 + 1delG)) (Table 1) [51]. A second reported case also confirmed that a patient was first diagnosed with MPNST, which led to a diagnosis of this patient’s family of being associated with BAP1-TPDS (pathogenic *BAP1* sequence variant c.122 + 1del) (Table 1) [52]. More recently, Rókusz et al. reported two patients with a newly described vulvovaginal epithelioid mesenchymal neoplasm with a “deciduoid” morphology and showed the loss of BAP1 protein expression and that they carried inactivating germline *BAP1* variants, c.784-1G > T and c.517T > G, respectively [53]. These findings further expand the spectrum of mesenchymal neoplasms associated with BAP1-TPDS. However, because this represents a newly proposed pathologic entity and none of the four reported tumors recurre during the available follow-up, the current study does not definitively establish whether these BAP1-associated neoplasms should be classified as malignant sarcomas or another specific cancer type. These recurrent cases suggest that although current screening recommendations for patients with BAP1-TPDS focus primarily on an annual physical examination, abdominal imaging, and ophthalmologic surveillance, emerging case reports indicate that additional tumor types, including MPNST, may occur in this syndrome. These observations suggest that the clinical spectrum of BAP1-TPDS may be broader than currently recognized and could inform the future refinement of surveillance guidelines and targeted therapeutic strategies for syndrome-associated MPNST.

**Figure 3:**
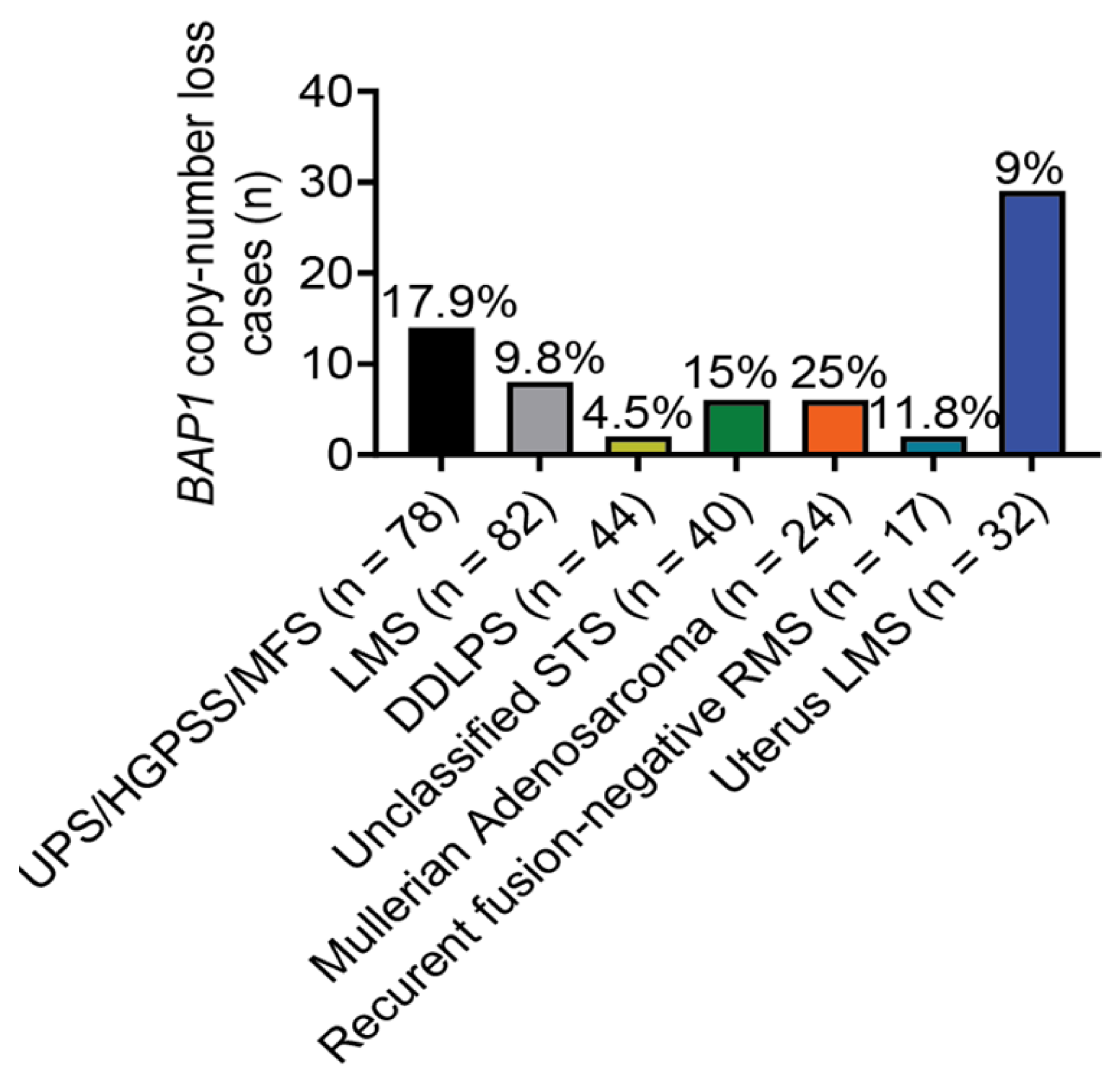
Distribution of *BAP1* copy-number loss across STS subtypes in the sarcoma cohort. Bar graph showing the number of STS cases with *BAP1* copy-number loss among major sarcoma subtypes. *BAP1* loss was most frequently observed in Mullerian adenosarcoma, UPS/high-grade pleomorphic spindle cell sarcoma (HGPSS)/myxofibrosarcoma (MFS), and recurrent fusion-negative rhabdomyosarcoma (RMS), followed by leiomyosarcoma (LMS), uterus LMS, dedifferentiated liposarcoma (DDLPS), and other or unclassified sarcomas.

Mullerian adenosarcoma is a rare mesenchymal tumor arising from the gynecologic tract. A study showed that 25% of cases of high-grade mullerian adenosarcoma are associated with *BAP1* loss, suggesting that *BAP1* reduction is a potential driver of adenosarcoma (Figure 3) [54]. In this study, they expanded the examination of *BAP1* copy numbers in several subtypes of sarcoma and showed that *BAP1* copy number loss is also detected in endometrial stromal sarcoma, leiomyosarcoma, PEComa, RMS, and undifferentiated/unclassified sarcoma (Figure 3). Additional evidence linking *BAP1* to RMS comes from a sequential genomic study of paired primary and relapsed RMS samples. In the analysis of 35 paired RMS cases, including both fusion-positive and fusion-negative RMS, de Traux de Wardin et al. identified *BAP1* deletion among 2/17 of relapsed RMSs, supporting the possibility that *BAP1* disruption may occur in RMS, in addition to other sarcoma subtypes (Figure 3) [55]. Targeted NGS sequencing in one primary intracranial sarcoma (PIS) showed a *BAP1* p.S325F missense mutation (exon 11) [56]. Among uterus sarcomas, 1.4% of 509 cases harbor the deep deletion in *BAP1* [57]. Particularly, 9% of uterine leiomyosarcomas from that database harbor the deep deletion in *BAP1* (Figure 3).

Through additional multi-omics analysis of the TCGA database, Sturgill IR et al. found that 30/244 STS samples are all associated with the copy number loss of *BAP1* [51]. Among those samples with a *BAP1* deletion, 14 are from UPS/high-grade pleomorphic spindle cell sarcoma (HGPSS)/myxofibrosarcoma (MFS) (14/78). Eight are from leiomyosarcoma (LMS) (8/82). Two are from dedifferentiated liposarcoma (DDLPS) (2/44). The remaining six samples are from other or unclassified sarcoma (6/40) (Figure 3).

Although sarcoma has received relatively limited attention in BAP1 research, its absence or underrepresentation in major BAP1-TPDS cohorts does not necessarily preclude a biological association. This may partly reflect the rarity and heterogeneity of STS, limited disease-specific ascertainment, and the historical focus on more established BAP1-associated tumors, including mesothelioma, uveal melanoma, and renal cell carcinoma. Notably, the recent prospective study by Wu et al. also reflects the limited recognition of sarcoma within the BAP1 field. The authors noted that sarcoma had not been associated with BAP1 cancer syndrome, which was cited as part of the rationale for examining normal tissues, such as dermal fibroblasts, in their epigenomic analyses [58]. Together with emerging genomic, clinical, and functional evidence linking BAP1 loss to sarcoma, these observations support the greater consideration of STS in future BAP1-focused studies.

### 5.2. Functional Studies of BAP1 in Sarcoma

Functional studies of the underlying mechanisms by which BAP1 represses sarcoma initiation and progression are mainly in mesothelioma, uveal melanoma, renal cell carcinoma, cholangiocarcinoma, and other commonly associated cancers [4,26,33,35,44,59,60,61,62,63,64,65,66,67,68]. The first report showing that BAP1 regulates sarcoma was from Gao S et al. [69]. In this study, they demonstrated that the restoration of BAP1 suppressed osteosarcoma cell proliferation, migration, and invasion, while promoting apoptosis, supporting a tumor-suppressive role for BAP1 in osteosarcoma. Mechanistically, the authors linked these phenotypes to attenuation of the phosphoinositide 3-kinase/protein kinase B (PI3K/Akt) pathway, a central survival and motility-promoting signaling cascade in osteosarcoma. They further reported a physical association between BAP1 and microRNA 125 (miR-125), suggesting that BAP1 may constrain osteosarcoma progression at least in part through a miR-125-PI3K/Akt regulatory axis. Although this study provided initial in vitro evidence that BAP1 may suppress osteosarcoma cell growth and migration through a proposed miR-125–PI3K/Akt axis, the lack of in vivo validation and potential cell-line-specific effects limit the generalizability of these findings.

Building on these earlier observations, our recent study provided direct in vivo evidence that *Bap1* functions as a potent tumor suppressor in sarcoma. Using a tailored CRISPR/Cas9 screen in genetically engineered mouse models of UPS, we identified *Bap1* loss as a strong driver of sarcoma development, thereby linking BAP1 deficiency to the biology of complex-karyotype STS rather than only to isolated case reports or genomic associations. Importantly, sarcoma development in these models required concomitant *Trp53* loss, supporting cooperation between *Bap1* and *Trp53* loss, rather than demonstrating that *Bap1* loss alone is sufficient to initiate UPS. Integrative analyses, including RNA sequencing, multiplex immunohistochemistry, and flow cytometry, further showed that *Bap1*-deficient sarcomas acquire a markedly immunosuppressive tumor microenvironment, indicating that BAP1 loss may influence not only tumor-intrinsic growth programs but also antitumor immunity. Consistent with this model, BAP1 protein expression was reduced in human UPS, whereas polo-like kinase 1 (PLK1) expression was elevated. Functional studies demonstrated that PLK1 is required for the growth and survival of *Bap1*-deficient sarcomas, and pharmacologic PLK1 inhibition with volasertib suppressed tumor growth in both syngeneic and autochthonous mouse models. Importantly, combining PLK1 inhibition with anti-PD-1 therapy enhanced tumor control and improved survival compared with either treatment alone. Together, these findings establish *BAP1* loss as a functional driver of sarcoma and nominate PLK1 dependency and immune suppression as therapeutically actionable features of BAP1-deficient sarcoma [62,63].

However, the relationship between BAP1 and PLK1 appears to be highly context dependent. In contrast to our observation that *PLK1* is elevated and therapeutically required in BAP1-deficient sarcoma, studies on lymphoid development showed that BAP1 can occupy regulatory regions of cell cycle genes, including *PLK1*, and support their transcription through its H2AK119 deubiquitinase activity. In both B and T lymphocyte models, the loss of *BAP1* impaired cell cycle progression, reduced the expression of E2F/cell cycle programs including *Plk1*, and caused developmental arrest and/or apoptosis during lymphocyte maturation [70,71]. These findings suggest that BAP1 may positively regulate PLK1 in normal proliferating lymphoid cells, whereas BAP1-deficient sarcoma may acquire or select for PLK1 upregulation through distinct tumor-specific mechanisms. The mechanisms underlying this differential regulation of PLK1 by BAP1 across cell types remain unclear; one possibility is that additional *TP53* mutations in sarcoma induce broader epigenetic reprogramming, although this hypothesis requires further investigation. Thus, the BAP1–PLK1 axis should not be interpreted as a uniform linear pathway across tissues; rather, it likely reflects lineage-specific chromatin states, the developmental context, and tumor-adaptive dependencies.

Additionally, emerging evidence suggests that *BAP1* loss can influence the tumor immune microenvironment in sarcoma. In our recent study, RNA-sequencing analysis showed that *Bap1*-deficient sarcomas exhibited the downregulation of multiple immune-related pathways, including B-cell signaling, immune activation, and MHC class II-associated pathways. Multiplex immunohistochemistry and flow cytometry further demonstrated reduced T-cell infiltration and/or activity in *Bap1*-deficient tumors, including reduced CD3+ T-cell infiltration, decreased proliferation of CD8+ T cells, and an increased Treg-to-CD4+ T-cell ratio. *Bap1* loss was also associated with an altered myeloid composition, including increased PMN-MDSCs in the *Trp53/Rb1/Bap1*-deficient model compared with its *Bap1*-intact counterpart. Collectively, these findings support an association between BAP1 deficiency and an immunosuppressive tumor microenvironment in sarcoma, although the molecular mechanisms linking BAP1-dependent chromatin regulation to altered immune-cell recruitment and function remain to be determined [5,6,45,46,60]. Overall, the mechanistic study of BAP1 in regulating sarcoma initiation, progression, and immune suppression warrants future investigation.

## 6. Clinical and Therapeutic Implications

The emerging evidence linking *BAP1* alterations to sarcoma has several clinical implications. First, the *BAP1* status may define a biologically distinct subset of STS characterized by tumor suppressor loss, chromatin dysregulation, tumor immune microenvironment, and altered DNA damage responses [5,6,46,47,61]. At present, the prognostic significance of *BAP1* loss in STS remains incompletely established because most available data come from small case reports, subtype-restricted analyses, pan-cancer sequencing datasets, or preclinical models. Nevertheless, several observations suggest that *BAP1* deficiency should not be ignored clinically. *BAP1* copy-number loss is detectable across multiple sarcoma subtypes, including UPS/MFS, leiomyosarcoma, dedifferentiated liposarcoma, RMS, Mullerian adenosarcoma, and uterine sarcoma, and functional data support a tumor-suppressive role for BAP1 in both osteosarcoma and UPS [5,6,54,55,69]. These findings raise the possibility that *BAP1* loss may identify sarcomas with distinct patterns of progression, treatment vulnerability, or immune microenvironment remodeling, although larger subtype-matched clinical cohorts are needed to determine whether *BAP1* alterations independently predict survival, recurrence, metastasis, or the therapeutic response.

Second, BAP1 has potential value as a biomarker, but its assessment in sarcoma should extend beyond simple mutation calling. In STS, particularly complex-karyotype tumors, functionally meaningful BAP1 disruption may occur through copy-number loss, reduced protein expression, or downstream transcriptional and epigenomic consequences rather than recurrent hotspot mutations. Therefore, a practical biomarker strategy should integrate DNA sequencing, copy-number analysis, RNA expression, and BAP1 immunohistochemistry whenever tissue availability allows. Importantly, the *BAP1* status should also be interpreted in a histologic context. For example, the biological meaning of *BAP1* deletion in UPS may differ from that in uterine leiomyosarcoma, rhabdomyosarcoma, or gynecologic adenosarcoma. Subtype-specific validation is therefore essential before BAP1 can be used for routine risk stratification or treatment selection in STS. An important prerequisite for clinical translation is the establishment of standardized assays, scoring criteria, and validated cutoffs for defining *BAP1* deficiency in sarcoma. Clinical experience with BAP1 immunohistochemistry in other cancers, particularly mesothelioma, provides a useful methodological framework: the complete loss of tumor-cell nuclear staining is interpreted in the presence of retained nuclear staining in non-neoplastic cells, such as stromal or inflammatory cells, which serve as internal positive controls [72]. This pattern should be distinguished from reduced staining intensity or heterogeneous nuclear expression. For sarcoma studies, the fixation conditions, antibody selection, staining platforms, and scoring procedures should be standardized, with explicit reporting of the proportion, intensity, and spatial distribution of nuclear staining and assessment of interobserver and interlaboratory reproducibility. Similarly, copy-number analyses should distinguish single-copy loss from biallelic deletion while accounting for tumor purity and ploidy, and RNA-expression thresholds should be prespecified using platform-appropriate normalization and validated in independent cohorts. These complementary measurements should not be treated as interchangeable indicators of functional inactivation. Subtype-specific validation against genomic, functional, and clinical endpoints is therefore needed before BAP1-based classification can support routine risk stratification or treatment selection.

Third, *BAP1* deficiency may create therapeutic vulnerabilities that can be exploited in selected sarcomas. Because BAP1 participates in DNA repair and genome stability, BAP1-deficient sarcomas may respond to agents targeting DNA damage response pathways; however, these effects are likely to be subtype specific. Notably, our study showed that *Bap1*-deficient sarcoma was less sensitive to PARP inhibition in vivo, indicating that *BAP1* loss does not uniformly confer homologous recombination deficiency or PARP inhibitor sensitivity [15,49,64,65,66,67]. Accordingly, the activity of PARP, ataxia telangiectasia and Rad3-related protein (ATR), DNA-dependent protein kinase (DNA-PK), or other DNA damage response inhibitors should be evaluated in molecularly and histologically defined sarcoma models to identify context-dependent vulnerabilities and predictive biomarkers. Epigenetic therapy is another rational direction, given BAP1’s central role in the PR-DUB complex and H2AK119ub1 regulation. Strategies targeting enhancer of zeste homolog 2 (EZH2), histone deacetylases (HDACs), bromodomain proteins, or other chromatin regulators may be particularly relevant when *BAP1* loss leads to a Polycomb imbalance, chromatin compaction, or altered developmental gene expression [19,60,73,74,75,76]. Evidence from other cancers provides a mechanistic rationale for investigating epigenetic therapies in *BAP1*-deficient sarcoma. The loss of BAP1-mediated PR-DUB activity disrupts H2AK119ub1 homeostasis and can alter Polycomb-associated transcriptional regulation, although the resulting therapeutic dependencies are context dependent. In mesothelioma models, *BAP1* loss was associated with increased EZH2 expression and H3K27me3-mediated repression, and genetic or pharmacologic EZH2 inhibition suppressed *BAP1*-deficient tumor growth, supporting a vulnerability linked to altered Polycomb regulation [77]. HDAC inhibition offers a complementary approach to reversing BAP1-loss-associated transcriptional repression: in uveal melanoma, a functional rescue screen identified quisinostat, which reversed *BAP1*-deficiency-associated phenotypes and inhibited *BAP1*-mutant tumor growth in mice [78]. These findings support targeting the altered differentiation and transcriptional states associated with *BAP1* loss. A more direct link to H2AK119ub1 regulation has been demonstrated for bromodomain and extraterminal (BET) inhibitors in melanoma and clear-cell renal cell carcinoma models [76]. *BAP1* loss enhanced BET inhibitor sensitivity, whereas BAP1 deubiquitinase activity reduced sensitivity; conversely, expression of the H2AK119 ubiquitin ligases RING1A or RING1B increased sensitivity. Mechanistically, BET inhibition preferentially suppressed BRD4-dependent proliferative transcription, particularly MYC, in *BAP1*-deficient cells. Collectively, these studies connect BAP1-associated chromatin dysregulation to potentially targetable dependencies involving Polycomb repression, histone deacetylation, and transcriptional maintenance. Nevertheless, sarcoma-specific preclinical evidence remains sparse, and neither *BAP1* loss nor PR-DUB disruption alone should be assumed to predict sensitivity to these agents. To address this gap, we are systematically evaluating epigenetic inhibitors in *BAP1*-deficient sarcoma models to identify potential therapeutic vulnerabilities and determine whether the *BAP1* status influences treatment responses.

In addition, our recent in vivo CRISPR-based sarcoma study suggests a distinct therapeutic axis in which *BAP1*-deficient sarcomas acquire PLK1 dependency and an immunosuppressive tumor microenvironment; in that context, PLK1 inhibition with volasertib suppressed tumor growth, and the combination with anti-PD-1 therapy further improved tumor control [6]. These data support the broader principle that BAP1 loss may not be directly druggable, but it can generate secondary dependencies that are therapeutically actionable.

Finally, *BAP1* alterations have implications for genetic evaluations and surveillance. Although sarcoma is not currently considered a core tumor type of BAP1-TPDS, reports of leiomyosarcoma and MPNST in germline BAP1 carriers suggest that sarcoma may occasionally be part of a broader clinical spectrum [4,79,80]. For patients with unusual sarcoma presentations, multiple primary tumors, and a personal or family history of mesothelioma, uveal melanoma, renal cell carcinoma, characteristic melanocytic tumors, or early-onset malignancy, germline evaluations may be appropriate when a pathogenic *BAP1* alteration is detected in the tumor. Conversely, for known BAP1-TPDS carriers, clinicians should remain aware that rare mesenchymal malignancies may occur, even though available evidence is not yet sufficient to define sarcoma-specific surveillance guidelines. Overall, the clinical translation of BAP1 biology in STS will require the careful integration of genomic testing, protein-level validation, functional biomarkers, and prospective clinical correlations. Rather than treating BAP1 as only a pan-cancer tumor suppressor, future sarcoma studies should determine whether BAP1 loss identifies actionable biological states within specific STS subtypes. However, these therapeutic strategies are currently supported primarily by preclinical studies, and their clinical relevance remains uncertain, highlighting the need for further validation in *BAP1*-deficient sarcoma.

## 7. Future Directions

The study of BAP1 in sarcoma is still at an early stage. Although recent genomic and functional evidence supports *BAP1* loss as a driver in selected sarcoma subtypes, these findings represent a beginning rather than a complete mechanistic framework. A major priority is to define precisely how *BAP1* loss promotes sarcoma initiation, lineage transformation, progression, metastasis, and immune evasion. Future therapeutic development should proceed in parallel with mechanistic investigations. Because *BAP1* loss may generate different dependencies across histologic and molecular contexts, candidate therapies should be evaluated in well-characterized, subtype-specific preclinical models rather than assuming a uniform response across *BAP1*-deficient tumors. Functional screens and pharmacodynamic studies may identify actionable dependencies. Findings such as PLK1 dependency provide proof of principle, but additional studies are required to establish predictive biomarkers, rational drug combinations, and therapeutic windows before clinical translation. Patient-derived models and prospective molecular profiling will be important for determining which patients are most likely to benefit.

The possible sarcoma risk associated with germline *BAP1* pathogenic variants also warrants a systematic investigation. Sarcoma is not currently considered a core tumor type of BAP1 tumor predisposition syndrome, and available evidence is insufficient to support a dedicated sarcoma-screening protocol. Nevertheless, reported cases of leiomyosarcoma, MPNST, and others suggest that clinicians caring for germline BAP1 carriers should remain alert to persistent or enlarging soft-tissue masses and other concerning musculoskeletal symptoms. Future prospective registries of germline *BAP1* pathogenic variant carriers should capture the sarcoma incidence per carrier-year, histologic subtype, age and stage at diagnosis, diagnostic delay, and imaging-related false positives and downstream procedures. Such studies should evaluate symptom-triggered, site-directed imaging following clinical assessments of an unexplained enlarging soft-tissue mass, persistent focal pain, or new focal neurologic symptoms.

Additionally, future BAP1-focused sarcoma studies should incorporate age representation and equitable access into patient-derived model development. Collaboration across pediatric and adult sarcoma programs should support inclusion across the age spectrum, with age-stratified analyses accounting for the histologic subtype and treatment history rather than assuming that findings from adult tumors apply to pediatric disease. Prospective studies should also document access to molecular testing.

## 8. Conclusions

BAP1 is emerging as a tumor suppressor and potential biomarker in numerous sarcoma subtypes, e.g., eRMS, UPS, MFS, LMS, and MPNST. Although its mechanisms and clinical significance remain incompletely defined, BAP1 loss can promote sarcomagenesis and create context-dependent therapeutic vulnerabilities. Further mechanistic and clinical studies are needed to translate BAP1 biology into subtype-specific biomarkers, treatments, and surveillance strategies.

## Figures and Tables

**Table 1:** Reported soft tissue sarcoma cases associated with germline *BAP1* variants in BAP1 tumor predisposition syndrome. The table summarizes published sarcoma cases in individuals carrying germline *BAP1* variants, including the sarcoma subtype, reported germline variant, and corresponding reference.

Subtype	Germline *BAP1* Variant	Reference
vulvovaginal epithelioid mesenchymal neoplasm with “deciduoid” morphology	1 patient with c.784-1G > T, 1 patient with c.517T > G	Rókusz et al., 2026 [53]
Malignant peripheral nerve sheath tumor	c.122 + 1del	Cunliffe et al., 2025 [52]
Malignant peripheral nerve sheath tumor	c.659 + 1delG (IVS8 + 1delG)	Kaszuba et al., 2018 [51]
Leiomyosarcoma	c.1717delC	Testa et al., 2011 [4,42,43]

## Data Availability

This review summarizes previously published studies, which are cited in the manuscript. No original research datasets were generated for this work.
